# Supplementary material for: Association of trabecular bone score and bone mineral apparent density with the severity of bone fragility in children and adolescents with osteogenesis imperfecta: A cross-sectional study
Source: PLoS One. 2023 Aug 29;18(8):e0290812. doi: 10.1371/journal.pone.0290812 (PMC10464990; doi:10.1371/journal.pone.0290812)
Supplement: S1 Table — Age, age at the DXA scan; FR, the number of fractures up to the DXA scan divided by the age; BMDHAZ, height-for-age Z-score-adjusted bone mineral density-for-age Z-score; BMAD, Z-score of bone mineral apparent density; TBS, Z-score of trabecular bone score; Ht-SD, standard deviation score of height; BW-SD, standard deviation score of body weight; Tx, treatment at the DXA scan; past Tx, past treatment history; RIS, risedronate, PAM, pamidronate; ALN, alendronate; ZOL, zoledronic acid; Elde, eldecalcitol; Alfa, alfacalcidol; MSCT, mesenchymal stem cell transplantation in utero; none, no treatment; n.d., not detected; † We previously confirmed this deletion variant causing exon 21 skipping (p.Gly364_Arg399del) by mRNA analysis (Takeyari S, Kubota T, Ohata Y, Fujiwara M, Kitaoka T, Taga Y, et al. 4-Phenylbutyric acid enhances the mineralization of osteogenesis imperfecta iPSC-derived osteoblasts. J Biol Chem. 2021;296:100027. Epub 20201123. doi: 10.1074/jbc.RA120.014709. PubMed PMID: 33154166; PubMed Central PMCID: PMC7948972.). (DOCX) [file pone.0290812.s001.docx]

**S1 Table.** **Detailed data of the study participants**

|  | Sex | Sillence | Gene | variant effect | Age (yr.) | Fx | FR | BMD_HAZ_ | BMAD | TBS | Ht-SD | BW-SD | Tx | past Tx |
| --- | --- | --- | --- | --- | --- | --- | --- | --- | --- | --- | --- | --- | --- | --- |
| 1 | M | Ⅰ | *COL1A1* | c.3385C>T, p.Gln1129* | 4.99 | 2 | 0.40 | 1.12 | 1.33 | 1.46 | 1.01 | 1.72 | ALN | PAM, ALN |
| 2 | F | Ⅳ | *COL1A1* | c.1508G>C, p.Gly503Ala | 8.70 | 3 | 0.34 | 1.97 | 1.94 | 0.62 | -2.20 | -0.11 | PAM | PAM |
| 3 | M | Ⅰ | *COL1A1* | c.432delC, p.Gly145Aspfs*120 | 13.76 | 3 | 0.22 | 0.51 | 0.45 | 1.84 | -0.92 | -0.61 | RIS | PAM, RIS |
| 4 | M | Ⅰ | *COL1A1* | c.3112delG, p.Glu1038Argfs*70 | 20.39 | 4 | 0.20 | -2.34 | -2.49 | 0.42 | -1.00 | -0.13 | none | ALN |
| 5 | F | Ⅰ | *COL1A1* | c.2457dupT, p.Asp820* | 5.23 | 0 | 0.00 | -0.72 | -1.01 | 2.66 | -0.32 | 0.25 | none | none |
| 6 | M | Ⅴ | *IFITM5* | c.-14C>T | 13.85 | 5 | 0.36 | -2.48 | -3.00 | -0.62 | -1.94 | -0.97 | RIS | RIS |
| 7 | M | Ⅳ | *COL1A2* | c.2133+6T>A | 11.63 | 10 | 0.86 | 1.51 | 0.92 | 1.54 | -1.93 | 1.26 | PAM | PAM |
| 8 | M | Ⅰ | *COL1A1* | c.2715_2716dupCG, p.Gly906Alafs*203 | 14.10 | 2 | 0.14 | 0.48 | 0.46 | 1.26 | 0.14 | -0.57 | ALN | PAM, ALN |
| 9 | F | Ⅰ | *COL1A1* | c.2451+2T>G | 10.47 | 0 | 0.00 | 2.03 | 0.52 | 3.04 | -2.28 | -1.33 | RIS | PAM, RIS |
| 10 | M | Ⅰ | *COL1A1* | c.2451+2T>G | 12.65 | 2 | 0.16 | 0.22 | 0.37 | 2.91 | 0.03 | 0.00 | none | PAM |
| 11 | F | Ⅲ | *COL1A2* | c.1090-3_1155del, p.Gly364_Arg399del† | 13.00 | 21 | 1.62 | 5.41 | 1.29 | 1.49 | -7.49 | -1.39 | ZOL | PAM, ZOL |
| 12 | M | Ⅰ | *COL1A1* | c.2829+1G>A | 19.83 | 4 | 0.20 | -2.10 | -1.23 | -0.97 | 0.95 | -0.12 | Elde | RIS, Elde |
| 13 | M | Ⅰ | *COL1A2* | c.2314G>A, p.Gly772Ser | 9.99 | 6 | 0.60 | -0.39 | -0.36 | 0.82 | -1.69 | -0.47 | PAM | PAM |
| 14 | M | Ⅰ | *COL1A1* | c.2574delT, p.Pro859Leufs*249 | 12.81 | 4 | 0.31 | 1.09 | 1.24 | 0.55 | -1.23 | 0.11 | ALN | PAM, ALN |
| 15 | M | Ⅰ | *COL1A1* | c.862G>T, p.Glu288* | 19.61 | 6 | 0.31 | -0.03 | 0.70 | -0.01 | -0.04 | -0.39 | none | PAM, ALN |
| 16 | M | Ⅰ | *COL1A2* | c.1503+9_11delCAC | 14.39 | 5 | 0.35 | 0.77 | 0.37 | 1.49 | -1.75 | -2.92 | RIS | PAM |
| 17 | M | Ⅰ | *COL1A1* | c.2784delT, p.Gly929Alafs*179 | 7.11 | 5 | 0.70 | -2.53 | -2.14 | -0.56 | -2.39 | -3.89 | PAM | PAM |
| 18 | F | Ⅲ | *COL1A2* | c.3305G>T, p.Gly1102Val | 15.55 | 16 | 1.03 | 3.47 | 0.89 | -1.24 | -6.00 | -6.30 | ZOL | PAM, ZOL |
| 19 | M | Ⅲ | *COL1A1* | c.2299G>A, p.Gly767Ser | 9.58 | 12 | 1.25 | -0.57 | -1.14 | 0.81 | -4.16 | -2.06 | ZOL | PAM, ZOL |
| 20 | M | Ⅰ | *COL1A1* | c.3134delC, p.Pro1045Leufs*63 | 19.07 | 9 | 0.47 | 0.39 | 2.01 | -1.07 | 0.67 | 2.12 | none | PAM, ALN |
| 21 | M | Ⅰ | *COL1A1* | c.4249-33A>G | 17.82 | 6 | 0.34 | -0.52 | -1.17 | 0.40 | -2.41 | 0.00 | Elde | PAM, RIS, Elde |
| 22 | F | Ⅰ | *COL1A1* | c.441dupC, p.Gly148Argfs*21 | 7.49 | 1 | 0.13 | 2.59 | 2.07 | -0.65 | -1.76 | -1.27 | ALN | PAM, ALN |
| 23 | F | Ⅰ | *COL1A1* | c.1243C>T, p.Arg415* | 13.74 | 7 | 0.51 | 1.44 | 1.40 | -0.17 | -1.41 | -0.08 | RIS | PAM, RIS |
| 24 | F | Ⅲ | *COL1A1* | c.1515-2A>T | 12.01 | 16 | 1.33 | 2.43 | -2.21 | 0.52 | -7.87 | -3.70 | ZOL | PAM, ZOL |
| 25 | F | Ⅰ | *COL1A1* | c.3626_3635del, p.Ala1209Valfs*27 | 20.15 | 4 | 0.20 | -1.61 | -1.72 | -0.79 | -1.05 | -0.91 | Elde | Elde |
| 26 | F | Ⅰ | *COL1A1* | c.1299+2_1299+3insT | 19.16 | 15 | 0.78 | -1.41 | -0.91 | -2.23 | -0.10 | -0.09 | Elde | PAM, RIS, Elde |
| 27 | F | Ⅰ | *COL1A1* | c.387delT, p.Pro129Profs*136 | 19.47 | 5 | 0.26 | 0.24 | 0.76 | -1.23 | -1.24 | -1.18 | none | PAM, ALN |
| 28 | F | Ⅰ | *COL1A1* | c.3904C>T, p.Pro1302Ser | 18.85 | 1 | 0.05 | 0.91 | 0.64 | 0.52 | -1.18 | -0.46 | none | PAM, RIS |
| 29 | M | Ⅰ | *COL1A1* | c.2667+1G>C | 20.44 | 27 | 1.32 | -1.73 | -1.40 | -0.14 | -1.51 | -1.13 | none | PAM, ALN |
| 30 | M | Ⅰ | *COL1A1* | c.2235_2235+1del | 6.31 | 4 | 0.63 | -0.14 | -0.20 | -0.45 | -1.23 | 1.85 | PAM | PAM |
| 31 | F | Ⅳ | *COL1A1* | c.2461G>A, p.Gly821Ser | 10.74 | 2 | 0.19 | 3.06 | 1.68 | 1.99 | -2.90 | -1.00 | RIS | PAM, RIS |
| 32 | M | Ⅰ | *COL1A1* | c.3526_3530delCCTGT, p.Pro1176Trpfs*42 | 11.99 | 9 | 0.75 | 0.35 | 0.18 | -0.81 | -0.93 | -0.21 | RIS | RIS |
| 33 | F | Ⅲ | *COL1A1* | c.2461G>A, p.Gly821Ser | 10.60 | 18 | 1.70 | -1.45 | -3.64 | 1.03 | -4.85 | -3.81 | PAM | PAM |
| 34 | F | Ⅰ | *COL1A1* | c.2829+1G>A | 8.99 | 2 | 0.22 | 1.04 | -0.03 | -0.59 | -1.72 | -1.09 | none | PAM, RIS |
| 35 | F | Ⅰ | *COL1A1* | c.334-9A>G | 19.69 | 2 | 0.10 | -1.84 | -1.48 | -0.41 | -0.24 | -0.41 | none | PAM, ALN |
| 36 | F | Ⅳ | *COL1A2* | c.2774G>A, p.Gly925Asp | 13.44 | 4 | 0.3 | 3.28 | -0.60 | -0.37 | -7.87 | -5.21 | PAM | PAM |
| 37 | M | Ⅳ | *COL1A2* | c.2774G>A, p.Gly925Asp | 6.15 | 4 | 0.65 | 1.77 | 2.14 | -0.59 | -1.20 | -1.45 | PAM | MSCT, PAM |
| 38 | M | Ⅰ | *COL1A2* | c.395G>A, p.Arg132His | 19.68 | 8 | 0.41 | 0.42 | 1.29 | -0.37 | 0.18 | 0.52 | none | ALN, Alfa |
| 39 | F | Ⅰ | *COL1A1* | c.2347G>T, p.Glu783* | 13.82 | 3 | 0.22 | 1.46 | 0.87 | 0.87 | -1.88 | -1.49 | none | PAM, RIS |
| 40 | M | Ⅰ | *COL1A2* | c.2314G>A, p.Gly772Ser | 18.54 | 11 | 0.59 | 0.29 | 0.16 | -0.54 | -1.81 | -0.66 | none | PAM, RIS |
| 41 | F | Ⅰ | *COL1A1* | c.495T>G, p.Tyr165* | 17.93 | 6 | 0.33 | -0.05 | -0.08 | 0.53 | -1.10 | -2.10 | none | PAM, RIS |
| 42 | F | Ⅰ | *COL1A1* | c.3578_3655delinsTCATCAGCCG, p.Ser1193Ilefs*5 | 12.22 | 6 | 0.49 | -0.16 | -0.49 | 0.25 | -0.49 | 0.31 | PAM | PAM |

Age, age at the DXA scan; Fx, total number of fractures up to the DXA scan; FR, the number of fractures up to the DXA scan divided by the age; BMD_HAZ_, height-for-age Z-score-adjusted bone mineral density-for-age Z-score; BMAD, Z-score of bone mineral apparent density; TBS, Z-score of the trabecular bone score; Ht-SD, standard deviation score of height; BW-SD, standard deviation score of body weight; Tx, treatment at the DXA scan; past Tx, past treatment history; RIS, risedronate, PAM, pamidronate; ALN, alendronate; ZOL, zoledronic acid; Elde, eldecalcitol; Alfa, alfacalcidol; MSCT, mesenchymal stem cell transplantation in utero; none, no treatment; n.d., not detected;

† We previously confirmed this deletion variant causing exon 21 skipping (p.Gly364_Arg399del) by mRNA analysis [1].

1. Takeyari S, Kubota T, Ohata Y, Fujiwara M, Kitaoka T, Taga Y, et al. 4-Phenylbutyric acid enhances the mineralization of osteogenesis imperfecta iPSC-derived osteoblasts. J Biol Chem. 2021;296:100027. Epub 20201123. doi: 10.1074/jbc.RA120.014709. PubMed PMID: 33154166; PubMed Central PMCID: PMCPMC7948972.
