# Supplementary figures and images for: Association of trabecular bone score and bone mineral apparent density with the severity of bone fragility in children and adolescents with osteogenesis imperfecta: A cross-sectional study
Source: PLoS One. 2023 Aug 29;18(8):e0290812. doi: 10.1371/journal.pone.0290812 (PMC10464990; doi:10.1371/journal.pone.0290812)

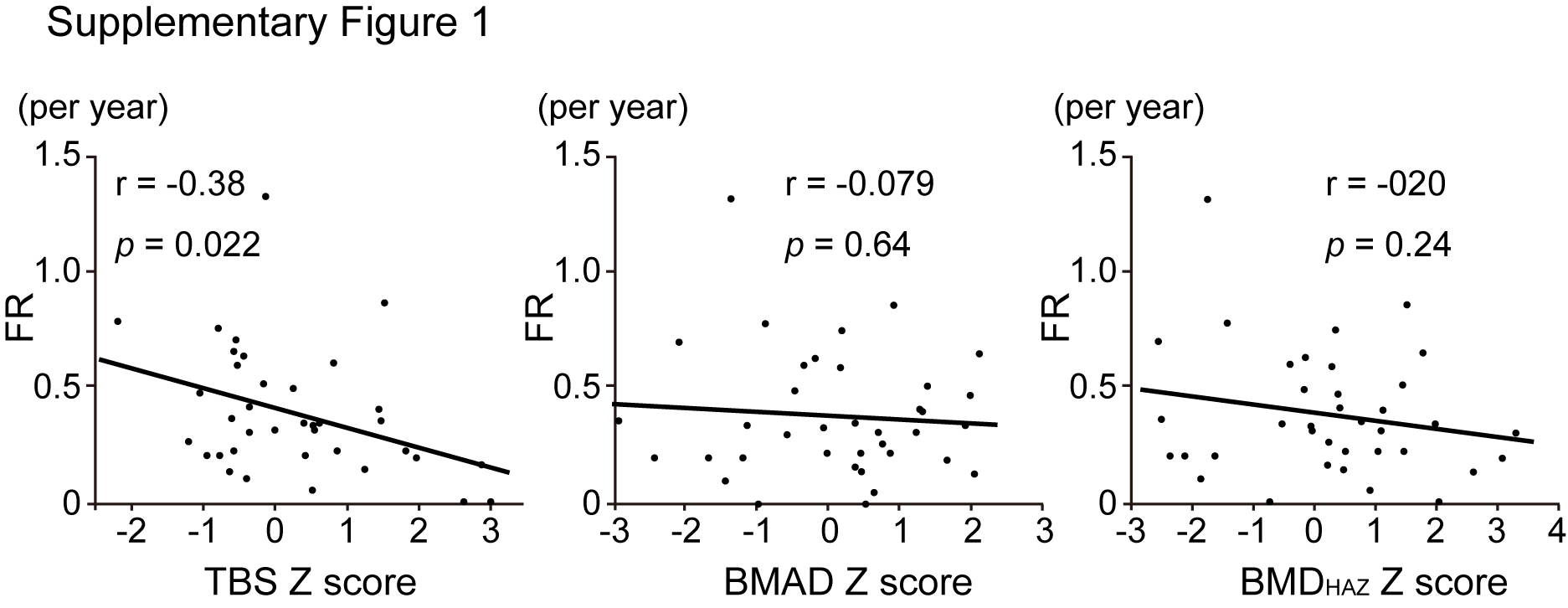

Supplement: S1 Fig — (TIF) [file pone.0290812.s002.tif]

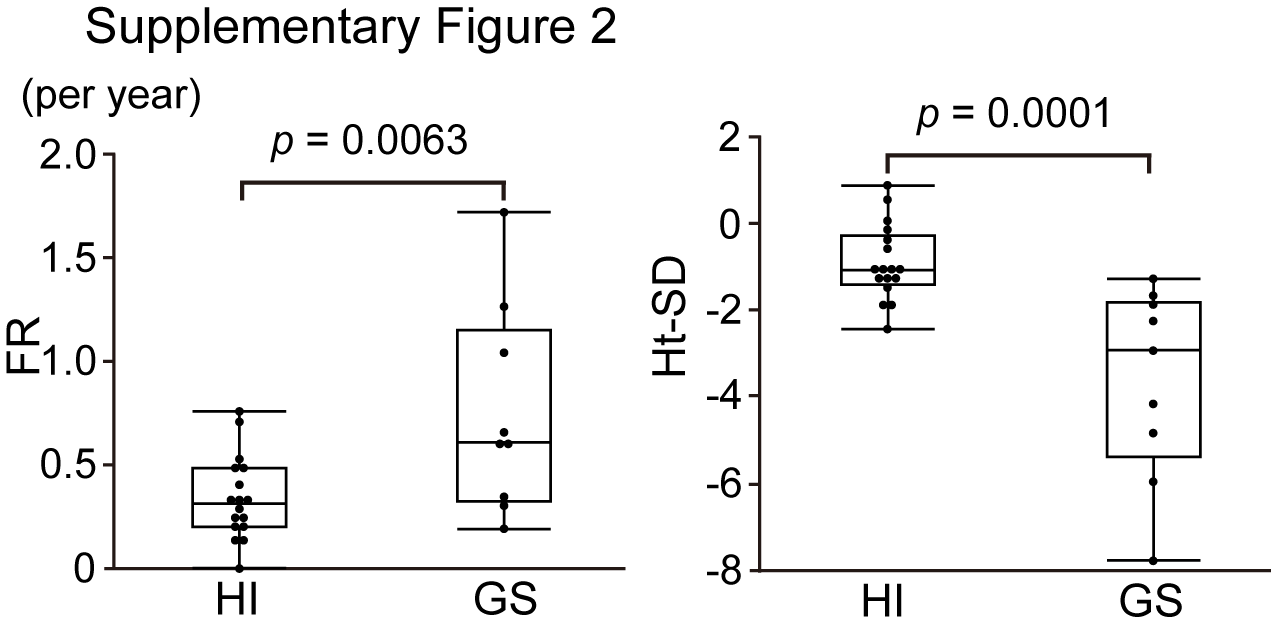

Supplement: S2 Fig — (TIF) [file pone.0290812.s003.tif]

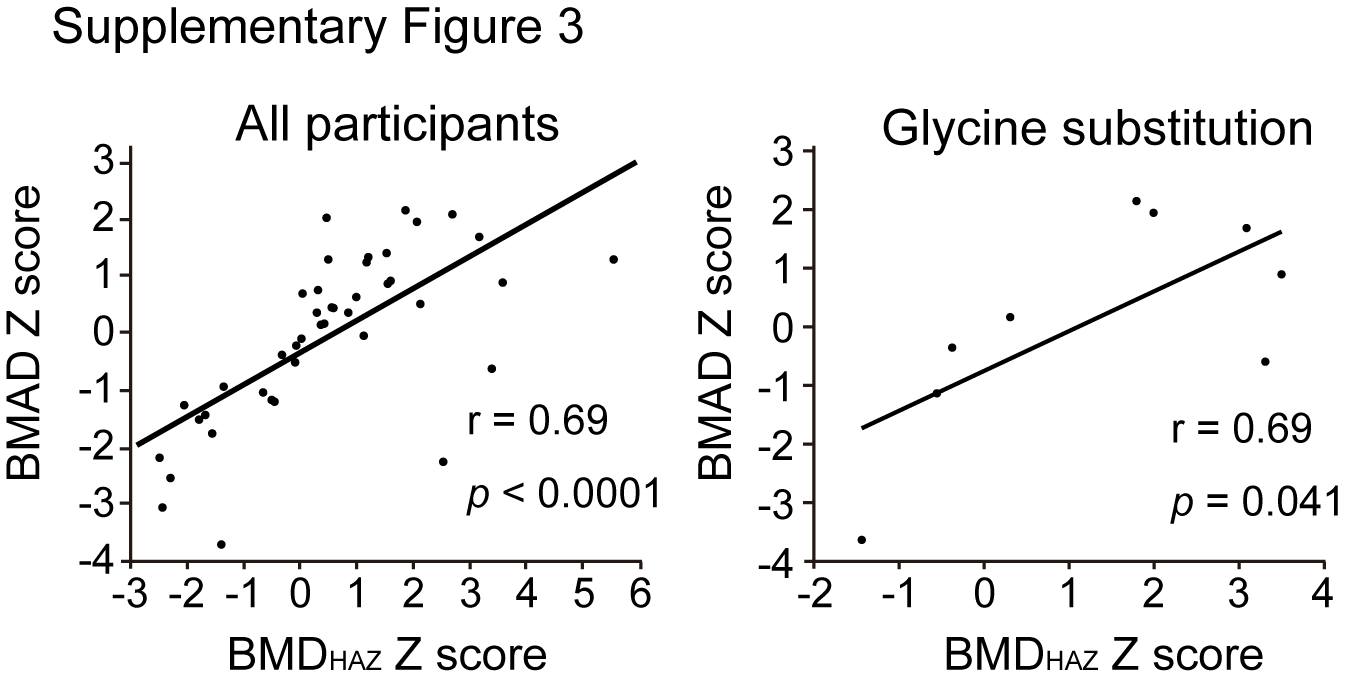

Supplement: S3 Fig — (TIF) [file pone.0290812.s004.tif]
