# Supplementary material for: Association of trabecular bone score and bone mineral apparent density with the severity of bone fragility in children and adolescents with osteogenesis imperfecta: A cross-sectional study
Source: PLoS One. 2023 Aug 29;18(8):e0290812. doi: 10.1371/journal.pone.0290812 (PMC10464990; doi:10.1371/journal.pone.0290812)
Supplement: Attachment — Submitted filename: Response to Reviewers.docx [file pone.0290812.s006.docx]

RESPONSE TO REVIEWERS

**Additional Editor Comments:**

**Thank you for addressing the reviewers' comments. Reviewer 2 has one comment on the Discussion: Page 18 end of paragraph 1: suggest removing “****likely due to the consideration of mechanical properties of the bone.” BMAD accounts for bone size but doesn’t specifically account for other mechanical properties of bone.**

**Response:** Thank you for your thorough review. As the Reviewer 2 has suggested, we have deleted the wording “likely due to the consideration of mechanical properties of the bone.” from the Discussion section.

**I have a few other minor comments:**

**1. In the abstract, start sentence 2 with** **Areal bone mineral density (BMD). Similarly, on page 5, add areal to the second sentence and remove "the" (****Evaluation of areal bone mineral density). Please also remove levels after "lower BMD" in this sentence. Further on in this paragraph, consider remove "of this" after "As a result" and remove the aBMD abbreviation after spine areal BMD.**

**Response:** Per your comments, we have started the sentence 2 with Areal bone mineral density (BMD) in the abstract. On page 5, we have revised the sentence to read “Evaluation of areal bone mineral density.” We have removed the words “levels” and “of this,” as well as the abbreviation “aBMD” from the specified location.

**2. End of pg 5/start of page 6: I find the sentence that begins with "Previously, two studies evaluated..." rather vague. Please reword (i.e., evaluated BMD?). Similarly, the last sentence of this paragraph (that begins with "Although both studies..." could be reworded - perhaps "Although neither study assessed bone fragility, BMD Z-scores and BMAD may be useful approaches to evaluate bone fragility in children and adolescents with OI" (or something like that!).**

**Response:** Thank you for your suggestion. To make it clear, we have changed the sentence to “Previously, two studies evaluated BMD in children and adolescents with OI using different parameters.” We also have changed the last sentence of this paragraph as you suggested “Although neither study assessed bone fragility, BMD Z-scores, and BMAD may be useful approaches to evaluate bone fragility in children and adolescents with OI.”

**3. Please change HRpQCT to HR-pQCT throughout the manuscript.**

**Response:** We have changed “HRpQCT” to “HR-pQCT” on page 6 and 17.

**4. Bottom of page 6: By "its widespread use" are the authors referring to HR-pQCT?**

**Response:** Thank you for your thorough check. We have intended for the term “widespread use” to specifically refer to bone biopsy. To make it clear, we deleted HR-pQCT from this sentence and reworded it as follows, “While the aforementioned findings provide the rationale for performing bone biopsy routinely in OI assessment of children and adolescents, this approach is difficult to implement given the invasiveness of bone biopsy and the specialized expertise required for its widespread use.”

**5. Discussion, first sentence: “density measures Z-scores of TBS…” could be reworded. In the 2^nd^ sentence, change “risk of fracture” to “fracture rate”. Please remove numerical results from the discussion – these should only appear in the results section.**

**Response:** Thank you for your advice. Following your suggestion, we have made two changes: firstly, the sentence now reads, “For the first time in existing literature, our study determined the correlations between Z-scores of TBS, BMAD, and BMD_HAZ_ and fracture risk in children and adolescents with OI.” Secondly, we have replaced “risk of fracture” with “fracture rate.” We have deleted the description of numerical results from the discussion section.

**Reviewers' comments:**

**Reviewer #1: All comments have been addressed adequately. The authors are congratulated on an interesting study. Always be careful when using DEXA to assess bones of different size!**

**Response:** Your careful and professional review greatly enhanced our manuscript. We deeply appreciate your invaluable advice and guidance.

**Reviewer #2: All comments have been addressed well.**

**One additional minor comment:**

**Discussion**

**Page 18 end of paragraph 1:**

**I suggest removing “likely due to the consideration of mechanical properties of the bone.”**

**BMAD accounts for bone size but doesn’t specifically account for other mechanical properties of bone.**

**Response:** Thank you for your suggestion. We have deleted the wording “likely due to the consideration of mechanical properties of the bone.” from the Discussion section. Your expert review has dramatically improved our manuscript. We sincerely appreciate your invaluable guidance.
